# Human Social Behavior and Demography Drive Patterns of Fine-Scale Dengue Transmission in Endemic Areas of Colombia

**DOI:** 10.1371/journal.pone.0144451

**Published:** 2015-12-14

**Authors:** Harish Padmanabha, Fabio Correa, Camilo Rubio, Andres Baeza, Salua Osorio, Jairo Mendez, James Holland Jones, Maria A Diuk-Wasser

**Affiliations:** 1 Centro de Investigaciones en el Desarrollo Humano (CIDHUM), Universidad del Norte, Km 5 Via Puerto Colombia, Puerto Colombia, Colombia; 2 National Socio-Environmental Synthesis Center (SESYNC), University of Maryland, 1 Park Place, Suite 300, Annapolis, Maryland, 21401, United States of America; 3 Instituto Nacional de Salud de Colombia, Avenida/calle 26 No. 51–20 - Zona 6 CAN, Bogotá, D.C., Colombia; 4 Department of Anthropology/Woods Institute of the Environment, Stanford University, 450 Serra Mall, Building 50, Stanford, California, 94305–2034, United States of America; 5 Department of Ecology, Evolution and Environmental Biology, Columbia University, 1200 Amsterdam Ave, New York, New York, 10027, United States of America; 6 Department of Epidemiology of Microbial Diseases, Yale University, 60 College St, New Haven, Connecticut, 06520, United States of America; Institut Pasteur, FRANCE

## Abstract

Dengue is known to transmit between humans and *A*. *aegypti* mosquitoes living in neighboring houses. Although transmission is thought to be highly heterogeneous in both space and time, little is known about the patterns and drivers of transmission in groups of houses in endemic settings. We carried out surveys of PCR positivity in children residing in 2-block patches of highly endemic cities of Colombia. We found high levels of heterogeneity in PCR positivity, varying from less than 30% in 8 of the 10 patches to 56 and 96%, with the latter patch containing 22 children simultaneously PCR positive (PCR22) for DEN2. We then used an agent-based model to assess the likely eco-epidemiological context of this observation. Our model, simulating daily dengue dynamics over a 20 year period in a single two block patch, suggests that the observed heterogeneity most likely derived from variation in the density of susceptible people. Two aspects of human adaptive behavior were critical to determining this density: external social relationships favoring viral introduction (by susceptible residents or infectious visitors) and immigration of households from non-endemic areas. External social relationships generating frequent viral introduction constituted a particularly strong constraint on susceptible densities, thereby limiting the potential for explosive outbreaks and dampening the impact of heightened vectorial capacity. Dengue transmission can be highly explosive locally, even in neighborhoods with significant immunity in the human population. Variation among neighborhoods in the density of local social networks and rural-to-urban migration is likely to produce significant fine-scale heterogeneity in dengue dynamics, constraining or amplifying the impacts of changes in mosquito populations and cross immunity between serotypes.

## Introduction

Dengue Fever (DF) is the most prevalent human arboviral disease and estimates of its global burden continue to grow [1]. As a complex system, the macro-epidemiology of DF at city, regional and global scales derives from individual-level interactions between the virus’ two main hosts, *Aedes aegypti* mosquitoes and humans, which propagate the 4 serotypes of dengue virus. These are two host species whose activity patterns come together in the context of the domestic environment, which constitutes the main place of resting, feeding and development of both in urban areas. Studies overwhelmingly demonstrate that human dengue cases tend to reside in areas with transmission between residents and *A*. *aegypti* within a 100-200m radius[2–5]. Thus, the drivers and dynamics of dengue transmission in a group of houses represent a fundamental aspect of dengue epidemiology.

Heterogeneous and stochastic infectious contact between *A*. *aegypti* and humans generates daily variation in the force of dengue transmission that theoretically drives the course of epidemics [6, 7]. For example, previous work shows that because *A*. *aegypti* populations emerge from only one or a few houses in a given area[8–11], some households receive a disproportionate amount of bites from *A*. *aegypti* taking their first meal[12]. When a member of these households becomes infectious, they can thus infect large numbers of mosquitoes, leading to >80% of simulated secondary infections in a completely susceptible block[12]. Such localized amplification of virus, if they did occur, would likely constitute an important mechanism of dengue persistence, as the opportunity to infect distant populations greatly increases when a large number of hosts are simultaneously infected [13]. Determining whether or not such intense amplification events actually occur is also of practical importance, because house-to-house surveys of *A*. *aegypti* pupal density are one of the most widely available means for entomological assessment of local dengue risk[14].

We therefore need a better understanding of the drivers and dynamics of dengue transmission at daily timescales. Active virus in humans can be detected for roughly 5 days post-infection using a PCR assay[15, 16]. However, since dengue in endemic areas is mainly an asymptomatic childhood infection[17], a direct estimate of the daily force of infection would require taking almost daily blood samples from healthy children in their homes. Previous studies have found clustering of dengue infections within a 100-200m radius, but at temporal scales of weeks to months[3, 5]. The highest infection rate in a single cluster recorded in these studies comes from a rural village of Thailand, in which up to 65% of children in a 100m radius were found with evidence of recent dengue infection[5]. In this study, infection status was determined using a combination of serological and/or PCR methods, and summed over two sampling points 15 days apart [2, 3, 5]. Antibodies associated with recent infection may linger for months post-infection and epidemiological evidence indicates that they provide cross-protection to other serotypes for years [18, 19]. Thus, the available studies of house-level dengue transmission indicate that infection tends to cluster in neighboring houses, but they do not allow us to directly infer the intensity of transmission at daily timescales.

Amplification of dengue virus is related to the multiple ecological processes affecting the vectorial capacity of *A*. *aegypti*, such as water infrastructure, housing density, vector control interventions, climate variation, etc. However, in areas with high vectorial capacity and a long history of dengue transmission, the potential for amplification events will be constrained if human susceptibility to an invading serotype cannot recover fast enough after prior epidemics[20]. The net rate of susceptible replenishment depends on two counter-acting forces: (1) the frequency of viral introduction and further depletion of susceptibility and (2) the entrance of new susceptible residents. Both of these are heavily influenced by human social processes and may vary at small spatial scales in endemic cities. For example, migrants from non-endemic rural areas often concentrate in particular areas and affect the spatial patterns of urban diseases [21]. Another example is the capacity of humans, through routine urban activities, to propagate dengue infection between isolated *A*. *aegypti* populations within the city [22, 23]. Interactions between behavioral and demographic process determining net susceptible replenishment may therefore be important in driving heterogeneity in dengue dynamics.

In this paper we synthesize field surveys with simulation experiments to investigate the drivers of dengue transmission in a pair of city-blocks. First, we use pilot surveys of dengue virus in children in order to obtain point estimates of dengue viral prevalence in 2-block patches, located in the historically highest transmission neighborhoods of 3 highly endemic Colombian cities. This field setting, ecologically very different from the rural villages of previous cluster studies, evidenced tremendous heterogeneity in the daily force of transmission. While a multitude of conditions may permit low levels of endemic transmission, in one patch we found in 22 of 23 children living in two adjacent blocks simultaneously positive for dengue serotype 2 (DEN2). In order to explore the historical conditions that allowed for such explosive transmission during an inter-epidemic period with presumably high levels of immunity, we then constructed an agent based model of the dengue system in the patch with the highest transmission. The model was parameterized with data from our longitudinal surveys of *A*. *aegypti* production (2007–2009) in roughly 700 houses in the same neighborhood as the explosive transmission event was observed [12]. The model’s daily time step and 20-year simulation period allowed us to integrate the timescales of transmission and those of human susceptible replenishment, thereby highlighting the interactions between human and vector ecology that drive dengue transmission in a group of neighboring houses. In particular, we focused on 4 ecological processes whose rates vary widely at small spatial scales (house, block, neighborhood) in typical dengue endemic cities: migration, long distance social contacts that may lead to viral introduction, birth rate and *A*. *aegypti* production.

## Methods

### Ethics statement

The protocol used in this study was approved by the institutional review board of the Colombian National Institute of Health (INS). Written informed consent was obtained from the parents of all children prior to drawing blood samples for dengue virus. Blood was drawn only from assenting children, obtained in writing for children 11–15 years old and orally for children below this age group.

### Dengue virus surveys

We carried out a pilot study of dengue in order to obtain point estimates of the prevalence of dengue virus in children in clusters of houses with evidence of recent transmission. Study patches, defined as two adjacent city blocks (ranging 50–138 houses, 182–369 residents), were located in six neighborhoods where we had conducted prior longitudinal studies of *A*. *aegypti* production and household water management in the Colombian cities of Armenia, Bucaramanga and Barranquilla. Three of the neighborhoods (La Fachada, La Cumbre, Ciudadela 20 Julio) had the most DF cases (2004–2006) in each respective city, and the other three were respectively among the top four reporting neighborhoods. In collaboration with municipal health departments, we worked to continuously identify clinical dengue cases in children below 6 years old between August 2010 and March 2011. In order to detect early onset dengue cases, nursing students and health department personnel routinely visited houses, daycares and schools to inquire about children with fever. Within 24 hours of notification of a febrile child, health department physicians visited the child’s home, performed a clinical evaluation and took a blood sample in all suspect dengue cases. Blood isolates were sent to the National Virology Reference Laboratory in the Colombian National Institute of Health (INS) (Bogota) and recent dengue infection was confirmed using RT-PCR[15, 24].

Our research team (based in the INS) displaced to the study neighborhoods 1–4 weeks following laboratory confirmation of dengue and conducted a 1-time visit to all premises in the city-block of the case’s residence and in the block across the street. Blood was drawn from assenting children below 16 years of age with consenting parents. In each consenting residence, we inspected all water-holding containers and enumerated all *A*. *aegypti* pupae. We also conducted a demographic and behavioral questionnaire for descriptive purposes (see Table 1 and S1 File for more information).

**Table 1:** Frequency of dengue viremia in children and demographic features of each of 10 2-block study patches in highly endemic neighborhoods of Armenia, Barranquilla and Bucaramanga, Colombia. Additional information on *A*.*aegypti* habitats and visitors to homes provided in Table A in S1 File.

Cluster ID	City (neighborhood)	Children surveyed for dengue	rtPCR Positive (%)	No. houses (household size)	Population (%< 5 years old)	Residence in neighborhood (years)
**1**	Armeni (La Fachada)	23	22 (96%)	67 (4.0)	182 (14.8%)	6.3
**2**	Barranquilla(Ciudadela)	16	9 (56%)	58 (3.8)	223 (11.2%)	17.7
**3**	Armenia (Las Colinas)	28	8 (29%)	138 (3.9	312 (12.2%)	9.3
**4**	Barranquilla (Ciudadela)	14	3 (21%)	86 (4.3)	369 (8.4%)	15.0
**5**	Bucaramanga(La Cumbre)	25	4 (16%)	53 (5.0)	213 (11.3%)	22.5
**6**	Bucaramanga(Campohermoso)	43	6 14%)	64 (4.6)	223 (10.3%)	21.1
**7**	Bucaramanga(Campohermoso)	30	3 (10%)	79 (5.2)	261 (9.2%)	23.3
**8**	Barranquilla(Ciudadela)	10	0 (%)	114 (3.7)	222 (13.0%)	22.6
**9**	Bucaramanga(Campohermoso)	17	0 (0%)	50 (5.2)	215 (12.0%)	14.6
**10**	Barranquilla(Ciudadela)	20	0 (0%)	72 (3.0)	226 (8.6%)	17.8

### Viral RNA extraction and RT-PCR

Collected sera were used to extract viral RNA using QIAamp Viral RNA Minikit (Qiagen, Germany) following manufacturer’s instructions. RNA was finally eluted with 60 μl of AVE buffer and stored at -80°C until use. Five microliters from each RNA extraction were used as template in a one-step multiplex RT-PCR reaction (Qiagen, One-Step RT-PCR kit) as previously described [15]. Primers used were designated to amplify 751 bp from the E/NS1 region (primers available upon request). Reactions were tested for positive amplification in 1% agarose gel. Those negative samples in the first amplification round were then subjected to a nested PCR. The extraction processes was carried out in a different place than the PCR in order to limit any chance of contamination and cross amplification. In addition, negative controls including water instead of RNA were used to validate the results. Finally, amplified products (from RT-PCR or nested PCR) were purified and then used as template for sequencing reactions in order to determine the dengue serotype. The identity of the amplicons was confirmed by comparing each sequence using BLAST (Basic Local Alignment Search Tool). See S1 File for details on sensitivity and specificity of the laboratory protocol.

### Model description

#### Overview of modeling approach

**Fig 1:**
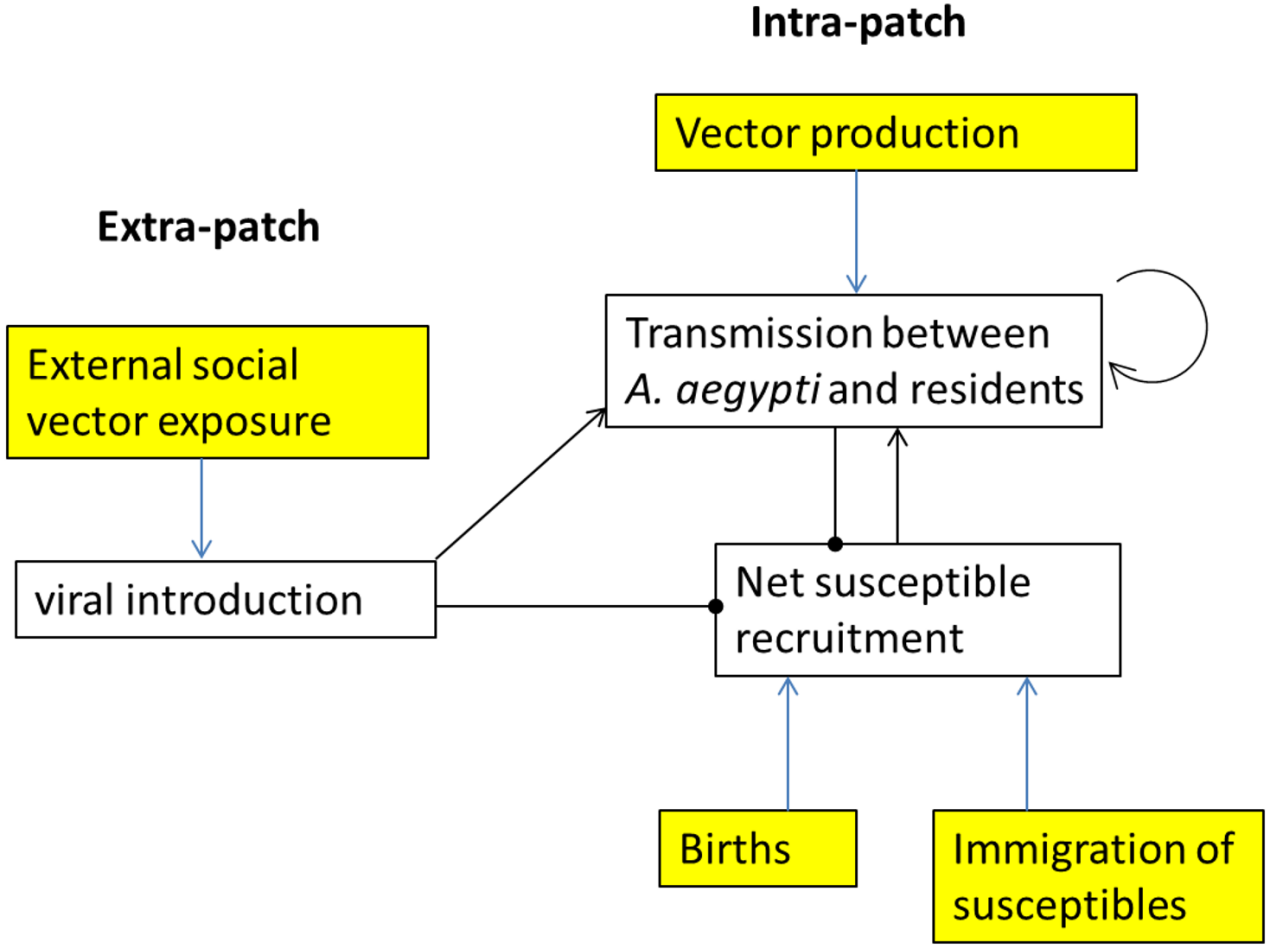
Schematic of dengue transmission system in an urban patch. Arrows represent causally increasing or self-enhancing effects, dots represent causally decreasing effects. For example, transmission between residents and mosquitoes on its own is self-enhancing because it amplifies the exposure of non-infected hosts to dengue. The transmission system is comprised of the interactions between the white boxes; yellow boxes are socio-ecological drivers of interest, represented as parameters in the ABM.

We used simulation as a tool to study the drivers and dynamics of a single dengue serotype over the course of 20 years (dt = 1 day) in a 2-block urban patch. Arboviral transmission contains many unknown variables relating to human, vector and viral ecology, whose interactions give rise to complex epidemiological dynamics. Given this reality and the small sample size of patches surveyed for dengue, we sought to improve our intuition of the historical interactions between social and ecological processes capable of generating the intense transmission observed in Armenia (see results). The simulation provides a more detailed understanding of how epidemiological dynamics vary with concomitant variation in the force of viral introduction from outside the patch, host-vector contact within the patch and recruitment of susceptible humans. The schematic in Fig 1 qualitatively summarizes the key relationships incorporated into our transmission system (black arrows/dots) and relates them to socio-ecological variables of interest (blue arrows).

#### Key assumptions and nuances

Perkins et al (2013) identify three salient complexities of mosquito-borne transmission systems essential to account for in order to understand the epidemic potential in a group of houses: (1) some people are exposed to more bites than others, (2) mosquitoes and hosts do not completely overlap in their spatial distributions (“poor mixing”) and (3) the density of susceptible hosts is finite [20, 25]. Multiple physiological and behavioral mechanisms may give rise to these complexities, many of which are not fully understood. Nonetheless, our ABM incorporates specific processes of human and *A*. *aegypti* ecology that previous studies suggest contribute to these features of dengue transmission in a group of houses. Heterogeneous exposure (Perkins’ feature 1) in the dengue system is driven by the fact that residents are more likely be exposed to *A*. *aegypti* bites than visitors[26], even though house-to-house visits between residents of dengue endemic cities are sufficiently frequent to play a role in transmission dynamics[22]. In our model residents have on average 5 times the likelihood of visitors to be bitten a mosquito in a particular house, consistent with previous models [27]. Poor mixing in dengue is driven by the overwhelmingly consistent finding that most *A*. *aegypti* emerge in only a few houses which change over time [8, 9, 11], a pattern that strongly influences transmission dynamics in a two-block patch [12]. Our model readily incorporates this feature by using house-to-house pupal counts from two-block patches collected over 2.5 years in the La Fachada neighborhood to parameterize the house of each emergent host-seeking *A*. *aegypti*. This feature, which can significantly affect transmission dynamics [12], sets our model apart from other simulations that assume mosquito recruitment to be spatially random, in equilibrium, aggregated over larger spatial scales or do not explicitly simulate mosquitoes [20, 23, 25, 27–29]. We incorporate Perkins’ third feature, finite host density, by modeling the counteracting forces that together determine susceptible dynamics in an area that has experienced prior epidemics: susceptible recruitment (birth, death and immigration of non-immunes) and viral introduction through either susceptible resident infection by external vector populations (not within the simulatation area) or infectious visitor transmitting to local *A*. *aegypti* populations. We focused analysis on exploring how the processes generating Perkins’ features 2 and 3 combine to influence daily dengue dynamics, mainly because of their strong potential to vary over time and at small spatial scales such as the block or neighborhood. Sensitivity analyses (see supplement) also considered interactions between Perkins’ features 1 and 3.

An increasing number of studies identify human exposure to *A*. *aegypti* bites in places where they do not reside as important to dengue transmission, leading to diverse approaches to incorporating human movement into dengue transmission models [23, 27, 30]. Intra-city movement is a basic social behavior that arises as the consequence of social relationships with individuals, groups or formal institutions, thereby shaping an individual’s time and space allocation in urban areas [31, 32]. Our focus on transmission in a two-block, purely residential area led to a natural distinction between modeling internal (contained within the patch) and external (outside the patch) social relationships. The latter lead to viral introduction into the simulation patch, whereas the former contribute to the force of transmission, although both internal and external social relationships generate differential mosquito contact between residents and visitors (Perkins’ feature 1). In our model mosquitoes bite in a single house in each timestep, as in other dengue models [6, 27], and disperse randomly across houses based on field estimates from an area of similar housing density in Rio De Janeiro [27, 33]. In order to compare model outputs with field observations, we explicitly tracked a PCR-positive state in humans. There is some evidence that PCR-positivity lasts longer than the duration of infectiousness to mosquitoes, and as such we assumed it was exponentially distributed with a mean of 7 days [15] (as opposed to 5 days for infectiousness[16]).

#### Spatial layout

The simulation patch consists of a 2-dimensional 67-house grid taken from the urban planning grid of the two blocks in Armenia in which 22 children were found simultaneously with virus, herein referred to as a “PCR22 event” (see results section). The simulation patch is surrounded by a buffer zone of two rows of houses that provides a potential refuge for emigrating mosquitoes and a source of immigrant mosquitoes. This allowed us to more realistically simulate transmission dengue dynamics in two blocks embedded in a larger urban area, eliminating any possible aggregation of humans, mosquitoes and/or infection that might arise as an artifact of hosts being confined in a 2-block area. Mosquito and human dynamics and behavior in buffer zone houses are parameterized identically to houses in the simulation patch, but outputs and analysis focus only the simulation patch.

#### Dengue introduction through socio-ecological contacts with external areas

Our approach to modeling external dengue introduction into the simulation patch considers the viral introduction rate a function of the level of dengue transmission in the surrounding city, the exposure of susceptible residents to external infectious *A*. *aegypti* populations and the exposure of infectious visitors to local *A*. *aegypti* populations. The probability of introduction is given by the equation *p*_*intro*_ = *B*_*t*_ * *p*_*esv*_, where *p*_*esv*_ is the probability of external*-social-vector* exposure and *B*_*t*_ is a time-varying index describing the spread of dengue across neighborhoods in the surrounding city. *P*_*intro*_ represents a Bernoulli probability applied twice in each house-day to describe (1) the probability that an infectious visitor is exposed to biting mosquitoes present in the house (see below and [12] for description of reduced visitor biting probability) and (2) the probability that a susceptible resident becomes exogenously infected. Note that if this second process is a success, then one susceptible resident in the house is infected at random, and if all residents are immune then only the first process (infectious visitor exposed to local mosquito bites) is applied. There is thus a reduced rate of dengue introduction with heightened local immunity. Introduction may also occur via the buffer zone (immigration of infectious mosquitoes, visits to buffer zone, bites on visiting buffer zone residents), but external introduction into the buffer zone occurs via human-mediated introduction as described above.

*P*_*esv*_ is a constant daily probability that represents the level of infectious *A*. *aegypti*-exposing social contact between a household and people that do not live in the same block. This contact may occur in either the house (exogenous visitor exposed to local *A*. *aegypti*) or in external areas (resident visits to an area exogenous to the model system). Variation in *p*_*esv*_ generally reflects the overall level of social isolation of the patch with respect to surrounding areas. Given the lack of data and the complex socio-ecological dynamics that may give rise to *A*. *aegypti* exposure outside of one’s block, we varied *p*_*esv*_ exponentially in order to study a wide range of dynamical behaviors of the model. *B*_*t*_ varies between 0 (cases are concentrated in a single neighborhood) and 1 (cases are perfectly evenly distributed across neighborhoods) and roughly correlates with time series of DF cases in Armenia (see supplement for details on calculation of Bt). The time-series of B_t_ is repeated for the calculation of *p*_*intro*_ in simulation years 11–20. We chose to use the geographic spread of dengue, rather than total cases or prevalence in Armenia, because the focal nature of dengue transmission [19, 34, 35] means that cases are rarely evenly distributed across the city. The spatial extent of transmission is thus a better indicator of risk than the total number of cases, as humans do not distribute their social behaviors evenly across the city. This is consistent with data from dengue endemic cities suggesting that humans visit only a few destinations further away from home and these vary stochastically over time[36].

#### Within-patch social interactions and human demography

In contemporary working class Colombian neighborhoods such as our study areas, people commonly socialize with neighbors on the street or on front porches. Children, in particular, freely play and explore the urban environment and enter friends’ houses. These social relationships develop more frequently with more immediate neighbors, with contacts often occurring between 1630 and 1930h, a period when *A*. *aegypti* are known to host-seek. These observations are consistent with theory on central-placed optimal foraging theory, which poses that the time and energy costs of more distant activity plays a major role in behavior and social relationships [37, 38]. Central place foraging theory is consistent with data from dengue endemic cities suggesting that humans preferentially carry out most life activites closer to home [36]. At the same time, the social relationships that people construct feedback and influence how and where people allocate time[39], a feature shown to be important in dengue transmission[23].

In order to account for socially-mediated exposure to *A*. *aegypti* bites in the immediate vicinity of an individual’s house, each human is potentially bitten by mosquitoes in a Poisson-distributed number of houses in the simulation patch each day (with a visitor’s probability of being bitten in each). In the absence of field data, we assume that on average children (2–15 years old) have double the *A*. *aegypti*-exposing visits of adults (mean of 3 and 1.5 visited houses/day, respectively). Each person randomly chooses a house to visit among a fixed set of contact premises. The probability that a person includes a premise in their contact set is defined by the sum of a distance-independent component, *link_p* (applied to all premises) and a distance-dependent probability, *link_n/d^*.*5* (applied only to houses on the same street), where *d* is the distance between the residence and the potential contact premise.

A number of our study neighborhoods are known destinations of migrants from non-endemic rural areas, which occurs through the occupancy of existing vacant houses (unlike other neighborhoods where settlers build new homes on unoccupied land). Accordingly, the model replaces residents of randomly selected houses with a completely susceptible household whose size and age structure are randomly drawn among households surveyed in previous studies in Armenia[12]. The range of annual replacement rates (*mig*) studied contains the inverse of the range of residence times observed in our demographic surveys in our dengue virus study (Table 1).

Initial household size and age structure in each house was taken from the demographic survey of the patch with highest observed transmission in Armenia (Table 1). Human birth rate and mortality, simulated by the random addition of a non-immune child (age 1) into a household, varies across a range that includes birth and age-specific death rates crudely estimated from Armenia’s 2005 census and 2009 vital statistics data. Given the short duration of the simulation in comparison to human life history, we did not expect human birth rate and mortality to strongly influence dynamics.

#### Vector recruitment dynamics

Newly emerged host-seeking mosquitoes randomly appear in each house at a Poisson distributed daily rate whose parameter is a function of the house’s pupal count, sex ratio, pupal survival rates and durations of the pupal state and refractory period (period between female emergence and receptivity to mating). Pupal counts are assigned from one of 8 different 2-block patches in the La Fachada neighborhood in which we conducted 7 seasonal *A*. *aegypti* surveys of each house between 2007 and 2009 [12]. The algorithm used to assign pupae from surveys seeks a balance between conserving the observed spatial (across houses) and temporal (across surveys) patterns of *A*. *aegypti* production, while allowing for potentially large and stochastic fluctuations in production due to household behaviors (see S1 File for supporting details). In all sensitivity analyses (see below) vector production was varied by choosing a different patch to drive mosquito dynamics. Roughly an order of magnitude (x10) separated the patches with the highest and lowest frequency of highly productive houses (see below). Details about the following processes can be found in[12]: mosquito dispersal, mosquito biting frequency and host preferences, transition from pupal to host-seeking stage and extrapolation of pupal densities in house-surveys with missing data.

### Simulation and analyses

#### Analysis of simulated 20-year epidemiological patterns in a two-block patch

In order to explore the fine-scale patterns of transmission we studied the frequency distributions of outbreak size generated by different parameter scenarios of the model. We limited our analysis to years 11–20 in order to focus on the situation in which the patch is embedded in an endemic neighborhood, as opposed to an area where dengue has recently invaded and the population is immunologically naïve. For infectious diseases in which it can be assumed that outbreaks are generated by independent introductions (i.e. no long-term persistence), comparison of the shape of the frequency distributions of outbreak size can indicate the explosiveness and the mechanisms that drive transmission in different areas or ecological conditions [40]. For example, in the power-law equation p(X = x) = x^-α^, where *x* is the size of independent outbreaks, an increased exponent *α* indicates a higher frequency of large outbreaks relative to small ones.

We fit the equation above to the frequency distribution of the total number of autochthonous infections (secondary and later generations) generated from each simulated introduction (by resident, visitor or mosquito). Distributions were defined by all independent introductions summed over 250 20-year runs of a given parameter scenario. In this analysis we jointly varied *p*_*esv*_ (0.001, 0.01, 0.1), *mig* (0, 0.08, 0.16) and vector production (based on observation in each of the 8 2-block patches followed 2007–2009). In each simulation mean annual birth rate varied randomly in the 0.5–0.20 interval, with random variation in each across household (+/- 0.05). We use Kolmogorov-Smirnov (KS) statistics to determine the best-fitting power-law exponent *a* for each parameter scenario. This procedure computes the maximal distance (D) between the cumulative distribution frequency (CDF) of the data (the agent based model results) and the fitted model (*x* ^*-a*^), based on the equation $D=max_{x>x_{min}}\left|S_{\left(x\right)}-P_{\left(x\right)}\right|$, where *S(x)* is the CDF of the model output and *P(x)* the CDF of the fitted model that best fit the data in the region *X>Xmin [41]*. Comparison of *D* across parameter scenarios provides a relative measure of how well they fit power law distributions, although it does not assign statistical significance to the parameter *α*. The exponent *α* gives a relative measure of the frequency of large relative to small outbreaks generated by a particular parameter combination.

#### Analysis of the likelihood of the observed high transmission event

In order to link the model to the dengue viral surveys, we evaluated the historical conditions that could potentially give rise to simulated PCR22 event (see results section). In order to determine how PCR22 events fit within the simulated epidemiological patterns we correlated the fit exponent *a* and the average number of PCR22 events in years 11–20, using the model outputs described above. A strong correlation between *a* and number of PCR22 events indicates that the latter are more likely to occur in historical conditions favoring larger outbreaks. The lack of correlation, by contrast, suggests that the occurrence of PCR22 events does not necessarily reflect long-term epidemiological patterns.

We also explored the frequency of PCR22 events across the simulated parameter space. In this analysis we used 1000 model runs for each parameter combination mentioned above, generating sufficient data to stratify outputs into categories of human birth and death rates (varied randomly across model runs). In order to evaluate how the drivers of PCR22 events evolve from the time when dengue is introduced into a completely naïve patch to an endemic situation we used classification and regression tree (CART) analysis to compare the effects of input variables on the average number of PCR22 events in the first 5 simulation years with years 11–20 (see S1 File for details on the CART methodology [42]). Because the regression trees depend entirely on the input parameters, some of which are arbitrarily defined, our goal was not to obtain predictive models for the frequency of PCR22 events, but to qualitatively explore the temporal changes in the topology of the best-fitting trees generated by a common set of parameters.

#### Effects of external social-vector exposure on sensitivity of PCR22 events to input parameters

Given our finding that the value of *p*_*esv*_ drove the dynamical behavior of the system at the space and timescales of study (see results), we then investigated how this variable conditioned the sensitivity of PCR22 events to each parameter in the model. For each of the three *p*_*esv*_ values we carried out a univariate sensitivity analysis of all parameters, varying each at 50%, 25%, 100%, 125% and 150% of its default value, with the exceptions of vector production and survival. A more conservative variation scheme was implemented for the daily survival probability of vectors, as a 25% reduction from the default value virtually eliminated all transmission (Table 2). Vector production was varied by choosing 5 of the 8 field patches, corresponding respectively to the 0, 25, 50, 75 and 100 percentiles in the frequency of containers with greater than 100 *A*. *aegypti* pupae. We measured the average number of PCR22 events (simulation years 11–20), tabulated over 200 20-year model runs for each parameter value, leaving all others at their default levels (Table 2).

**Table 2:** Model parameters. Default value of a parameter refers to median value in all model analyses, with random individual-level variation as indicated in Table. Exceptions are *mig*, *birth rate and vector production*, which were jointly varied in initial analyses (see methods section for values)

Parameter	Description	Default value (source)	Random variation across individual mosquitoes, humans or households: full width of rectangular distribution as a proportion of the default (median) value	Mean values for sensitivity analysis
IIP	Incubation time of Dengue fever in humans	5.75 days[20]	0.5	-50%, -25, 0, +25, +50%(+-50%)
Virem	Time that a person remains in the infectious state	4 days [6, 49]	0.5	+-50%
p_inf_mh_	Per-bite probability of transmission from mosquito to person	0.75[6, 49]	0.4	+- 50%
p_inf_hm_	Per-bite probability of transmission from person to mosquito	0.75[6, 16]	0.4	+-50%
link_p	Bernoulli probability distance-independent component for choosing homes for the permanent set of contacts	0.15 [specified]	1.33	+-50%
link_n	Bernoulli probability constant for choosing homes for the permanent set of contacts	0.2 [specified]	None	+-50%
bit_vis	Bernoulli probability weight of a mosquito biting each visitor	0.2 [26, 27]	None	+-50%
visit_y	Rate parameter of the Poisson distribution of number of within-block visits that a young person does in each day	3 visits/day (specified)	0.5	+-50%
visits_a	Rate parameter of the Poisson distribution of number of within-block visits that an adult person does in each day	1.5 visits/day (specified)	0.5	+-50%
t_m	Incubation time of Dengue fever virus in mosquitoes	12 days [16, 50]	0.5	+-50%
bite_max	Number of bites a mosquito can inflict each day	10 bites (calibrated to [29, 51])	0.5	+-50%
p_full	Bernoulli probability of a mosquito getting full at each bite	0.375 (calibrated to 29,49])	2/3	+-50%
Disp	Standard deviation of the Gaussian flight distribution	2.9 (calibrated to [33])	0.5	+-50%
Survival	Bernoulli probability of mosquito survival each day	0.84 [33]	0.1	0.72, 0.78, 0.84, 0.90, 0.96
Refr	Post-emergence pre-mating refractory period in which mosquito does not host seek or disperse	2 days (iestimated from [52])	None (determines Poisson emergence rate parameter)	+-50%
disp_emerg	Additional # refractory days mosquito can disperse before becoming host-seeking	1 day (estimated from[52])	None	+-50%
pup	# days until pupal emergence in Armenia (field measurements, water temperature 21–22°C)	2.7 days(field measured)	None (determines Poisson rate parameter)	Not included in sensitivity analysis
s_pup_	Fraction of pupae that survive	0.94 (field measured)	None (determines Poisson emergence rate parameter)	Not included in sensitivity analysis
Vector production	ID number of study patch used to input vector recruitment, based on frequency of vessels with >100 *A*. *aegypti* pupae	25 (0.44 vessels/day with >100 A. aegypti pupae, field measured)	None	27, 26, 25, 24, 22
*mig*	Avg. rate at which new susceptible households replace existing ones	1/9 per household*year (based on field data)	None	+-50%
birth rate	Annual birth probability per inhabited home.	0.15 (based on Armenia vital statistics)	1	+-50%

## Results

### Field observations of dengue infections in 2-block patches of highly endemic neighborhoods

Between August 2010 and April 2011 we searched for dengue virus in children living in 10 2-block patches containing the home of a dengue case (Table 1) located in six of the highest transmission neighborhoods of Colombia. All children testing positive must necessarily have become infectious within the 5–7 day window of detectable virus using the rtPCR assay [16]. We found large variation in infectious rates among patches. In 8 of 10 patches less than 30% of children surveyed were PCR+, with no virus detected in 3 patches. In one patch (Ciudadela, Barranquilla), however, we found 9 of 16 children and in another (La Fachada, Armenia) 22 of 23 children with detectable virus (Table 1). No other clinical dengue cases were reported in the La Fachada neighborhood at the time of the survey. All PCR+ children in all patches were infected with serotype 2 (DEN2). Overall, parents/caretakers reported a recent fever in 11 of the total 55 PCR+ children, yielding an 80% asymptomatic rate. Socio-demographic and entomological variables recorded can be found in Table 1 and the S1 File. Distinguishing features of the Armenia patch with high infection rate include the highest fraction of the population less than 5 years old (Table 1, Figure C in S1 File), the shortest household residence time in the neighborhood (Table 1) and highest number of *A*. *aegypti* habitats per house (Table A in S1 File). However, given the stochastic nature of many processes involved in the transmission cycle, such as viral introduction, host-vector contact, mosquito survival and transmission, among others, we did not consider it possible to infer the drivers of variation by evaluating statistical predictors in only 10 patches. Nonetheless, our finding demonstrates the potential for highly intense and focalized dengue transmission.

### Simulated dynamics of dengue transmission in a two-block patch

In order to explore the potential drivers of such intense transmission in a highly endemic area, we carried out simulation experiments using 288 different combinations of external social-vector exposure, immigration, birth rate and vector production (250 replicates each). We isolated endemic dynamics by running each simulation for 10 years prior to analysis in order to ensure ample opportunity for dengue to invade the patch and generate a background level of immunity reflective of the social and ecological conditions of each parameter combination. Overall, we found that transmission intense enough to generate at least 22 residents simultaneously PCR positive during years 11–20 (PCR22 events) occurred in only about 1 out of every 6 trials (16.7%). Even in the parameter scenarios most conducive to explosive epidemics there was on average less than one PCR22 event per replicate.

**Fig 2:**
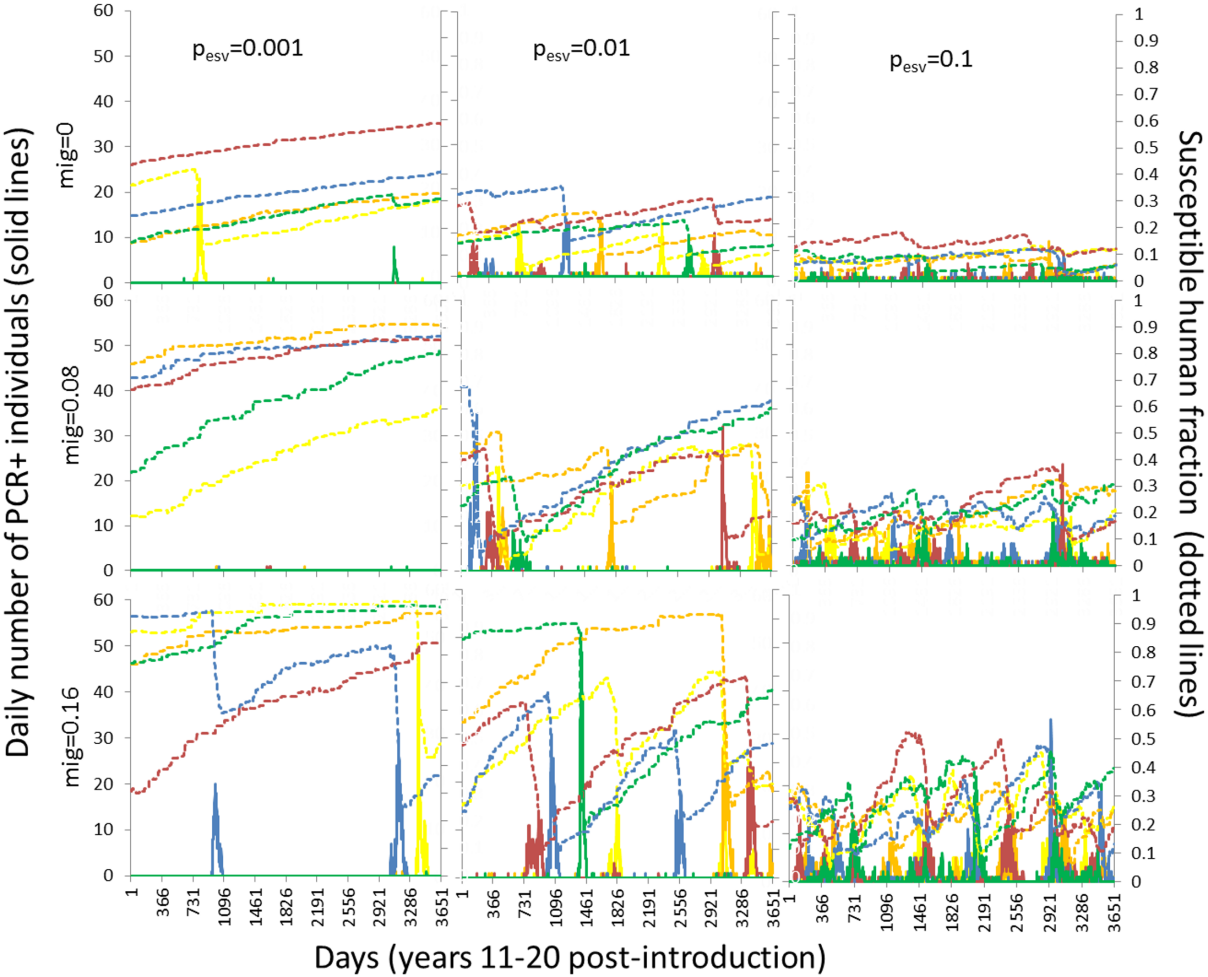
Daily dynamics of transmission 11–20 years post invasion for five randomly selected trials for each combination of external social vector exposure (*p*_*esv*_) (increasing from left to right) and migration (*mig*) (increasing from top to bottom. Birth rate was held at a constant probability of 0.15 births/household/year and vector production was input using the field patch with the highest average rate of A. aegypti production. Dotted lines track the fraction of the population susceptible to dengue (S) and solid lines track the number of infectious people (I).

Overall transmission dynamics showed substantial stochastic variation in the appearance of outbreaks, particularly under conditions permitting susceptibility to build up between introductions (low-intermediate *p*_*esv*_, higher immigration). Under these conditions we observe high variability in the initial susceptible fraction between replicates of the same parameter scenarios (Fig 2), indicating variation in the timing and/or magnitude of initial epidemics during the first 10 years. At low *p*_*esv*_ (Fig 2a,2d and 2g) the system was limited by lack of viral introduction rather than by the density of susceptible hosts. Many replicates at *p*_*esv*_ = 0.001 did not see any secondary transmission, as on average multiple introductions are required to spark an epidemic, even when the population is completely susceptible [12]. However, this allowed sufficient susceptible densities so as to maintain the potential for localized transmission of the intensity observed in La Fachada (Fig 2g). A qualitatively different transmission dynamic emerged when external social relationships generated constant viral introduction (*p*_*esv*_ = 0.1, Fig 2c,2f and 2i). In this condition susceptible residents more frequently became infected in external areas, which combined with an increased frequency of infectious visitors, resulted more opportunity for autochthonous local transmission. This ultimately limited the possibility for explosive outbreaks, as susceptibility among residents remained persistently low during the 10 years. The role of susceptible density in limiting transmission at *p*_*esv*_ = 0.1 is evidenced by the fact that moderately explosive outbreaks begin to stochastically emerge when immigration rates rose to 0.16 households/year (average of 1 non-immune family replacing a resident household every 6 years) (Fig 2i). Intermediate levels of external social vector contact (*p*_*esv*_ = 0.01) generated a balance between sufficient viral introduction and sufficient time for susceptible populations to recover between introductions, increasing the frequency of explosive outbreaks over the course of the simulation (Fig 2b,2e and 2h).

**Fig 3:**
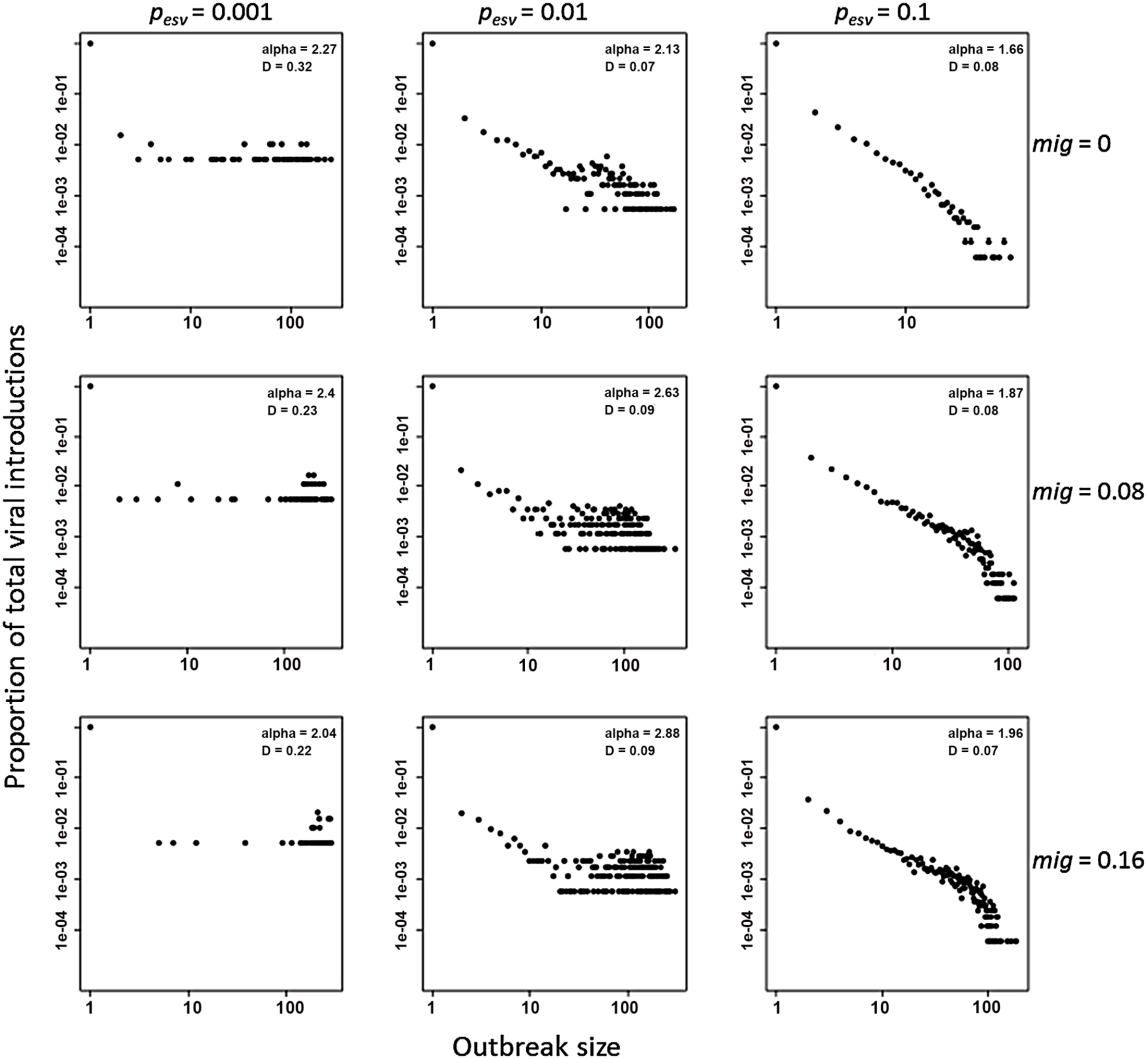
Simulated outbreak size distributions for each combination of *p*_*esv*_ and *mig* as shown in Fig 1. Each point represents an independent viral introduction into the 2-block patch in years 11–20, tabulated across 250 simulations for each parameter combination. Power law fits are shown in red. Lower *D* values indicate a better fit of the power law to the data. Alpha refers to the power law exponent (*a*). Vector recruitment is parameterized from the patch with the highest frequency of containers >100 *A*. *aegypti* pupae.

The histograms of outbreak size per viral introduction (Fig 3) further demonstrate the importance of social relationships with external areas in driving the general patterns of transmission. The tails of the distributions emerge at a frequency that varies inversely with *p*_*esv*_, suggesting that larger epidemics are proportionally more frequent in patches that are more socially isolated from surrounding areas. In other words where viral introductions are less frequent, they are more likely to generate explosive epidemics because of perpetually high susceptible densities (Fig 3a,3d and 3g). By contrast, social behavior causing frequent viral introduction (*p*_*esv*_ = 0.1) truncates the tail of the distribution as it approaches 100 infections. At *p*_*esv*_ = 0.1 the constraints on susceptible density did not allow the emergence of the “fat tailed” [40] distributions observed at *p*_*esv*_ = 0.01 and nearly bimodal distributions at *p*_*esv*_ = 0.001 (large outbreaks more proportionally frequent than small/medium-sized outbreaks). Analysis of power law fits confirm that simulated PCR22 events occur more frequently under parameter scenarios conducive to outbreak size distributions with fatter tails (see S1 File for details). Unlike external social-vector contact, increased immigration of non-immune households had little effect on the overall shape of outbreak size distributions, despite increasing the frequency of larger outbreaks. *P*_*esv*_ also modified the effects of variation in most model parameters on PCR22 frequency (see S1 File for details on sensitivity analyses).

**Fig 4:**
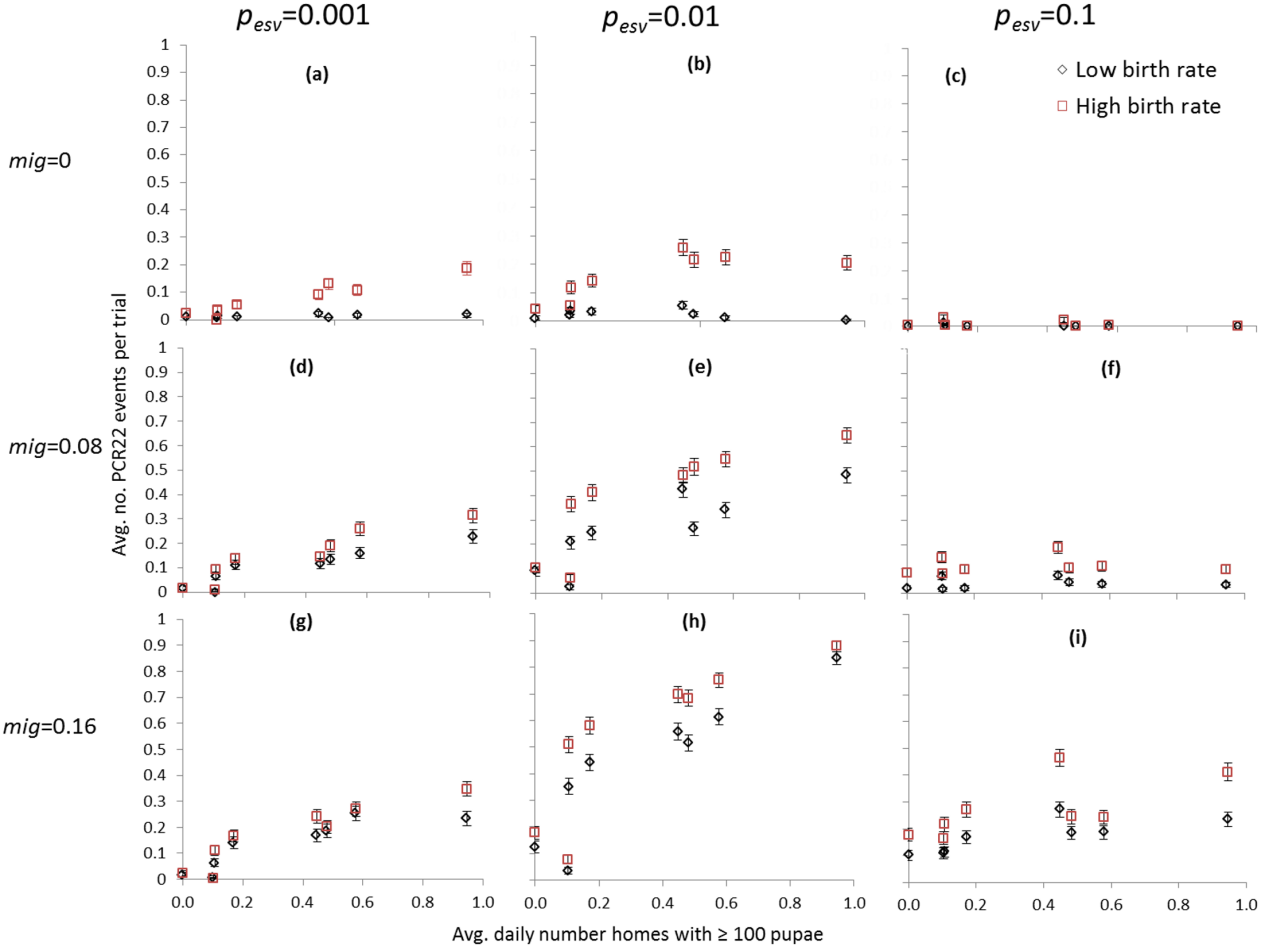
Number of PCR22 events 11–20 years post invasion (y-axis), averaged across 250 trials (s.e.) for each combination of *p*_*esv*_ and *mig* as shown in Figs 1 and 2. Birth rate averages 0.05–0.1 annual births/house in the low group and 0.2–0.25 annual births/house in the high group.

Overall, PCR22 events occurred much more frequently at intermediate rates of viral introduction through external A. aegypti-exposing social relationships (*p*_*esv*_ = 0.01, as compared to *p*_*esv*_ = 0.001 and *p*_*esv*_ = 0.1). Increased vector production also produced larger increases in PCR22 occurrence at intermediate as compared to low *p*_*esv*_. At high viral introduction (*p*_*esv*_ = 0.1), however, there was very little correlation between increased vector production and PCR22 events. For example, a roughly 10-fold increase in the frequency of mosquito habitats producing at least 100 *A*. *aegypti* pupae produced a similar 10-fold increase in PCR22 occurrence at *p*_*esv*_ = 0.01 (Fig 4e, intermediate immigration and high fertility), but produced no increase at *p*_*esv*_ = 0.1 (Fig 4f). Susceptible recruitment played an important role in PCR22 occurrence, with immigration of non-immune households having a much larger impact than fertility, due to the number of new susceptibles entering the simulation and their clustering in the same house. This is evidenced in the diminishing effects of increased fertility on PCR22 occurrence as immigration rates increased (Fig 4). Nonetheless, both immigration and heightened birth rates both magnified the effects of vector production on PCR22 occurrence. Regression trees showed that these interactions between external social relationships, immigration of non-immune households and of the frequency of PCR22 events are specific to endemic dynamics, as PCR22 events during initial invasion (Years 1–10) occurred frequently under most experimental conditions (see S1 File for more details).

## Discussion

Human social behavior and demography are known to influence the epidemiological patterns of dengue at scales of 100s to 1000s of meters and beyond, but their interactions with household level variation in host-vector contact are not well understood. Studies clearly demonstrate that dengue transmission occurs within and between neighboring houses [2–5], but also that immunity deriving from historical epidemiological patterns can drive local dynamics [19]. The urban block constitutes an important unit of dengue transmission because it is at this scale at which human demography and social relationships with the rest of the city intersect with three sources of heterogeneity [25] that determine the local potential for dengue transmission: differential exposure of residents and visitors to mosquito bites[26], aggregation across houses in the emergent vector population[12] and finite densities of susceptible hosts[19]. However, because most dengue infections are sub-clinical, knowledge of the history of block-level transmission is very difficult to obtain. Our overarching aim was to develop a better understanding of the patterns and processes of dengue transmission in pairs of city blocks, located in some of the highest transmission neighborhoods of Colombia.

We demonstrate the potential for substantial heterogeneity in the intensity of block-level dengue transmission. Based on the relatively low average daily rates of transmission that can be inferred from prior studies on fine scale dengue transmission[3, 4], there was little expectation of finding high levels of PCR positivity within city blocks, particularly in highly endemic areas of cities that have experienced prior of epidemics of DEN2. Indeed, in 8 of the 10 study patches PCR positivity in children was below 30%. Thus our findings of 9/16 PCR positive children in Barranquilla and 22/23 in Armenia confirm the theoretical prediction of high variability in the daily force of dengue infection [7, 12]. Given the short time window in which PCR positivity can be maintained, these data suggest an intensity of fine-scale dengue transmission far exceeding previous estimates from rural and urban areas. Particularly extraordinary is the simultaneous infection with DEN2 virus of 22 children residing in a 2-block patch in La Fachada, the highest DF-reporting neighborhood in Armenia (2001–2011). Armenia, in turn, was the Colombian city with the highest number of reported DF cases between 2001 and 2006, and almost certainly experienced epidemics of DEN2 during this period, based on INS records. The PCR22 patch had a number of distinguishing factors that might make it prone to dengue transmission, including the highest under five population, lowest residence time and highest Breteau Index (*A*. *aegypti* habitats per house). This finding begged the question of how susceptibility to DEN2 was maintained despite such intense historical dengue transmission in the surrounding areas.

Our ABM allowed us to address this question by integrating the timescales of epidemics (days, weeks) with the timescales of recovery of susceptibility at the patch level (years, decades). Overall, we found that social relationships exposing residents to *A*. *aegypti* bites outside the patch determined both the density of susceptibles and the impact of *A*. *aegypti* pupal production on endemic transmission. While susceptible recruitment through net immigration of non-immune households and high birth rates increased the amplitude of local epidemics, these did not qualitatively influence the patterns of transmission because their affect was unidirectional. By contrast, external A. aegypti-exposing social relationships have multiple opposing effects due to the negative feedback created by susceptible depletion (Fig 1). On one hand, resident exposure to infectious *A*. *aegypti* outside the patch provides an epidemic “spark”[40]. Because of both heterogeneous host-vector mixing[8, 26] and exposure [26] in dengue system, multiple sparks are necessary to ignite epidemics[12], and accordingly there was a strong increase in the frequency of PCR22 events when p_esv_ was increased by an order of magnitude from 0.001 to 0.01. However, a further increase from 0.01 to 0.1 meant that over time there were too many susceptible individuals being exogenously immunized and thus the mosquitoes they infected did not bite enough susceptible hosts to generate epidemics. Simulated epidemics were unpredictable in both timing and magnitude, but clearly increased with the combination of many highly productive vessels and increased susceptible densities. This result underscores the importance of spatial aggregation in pupae as a driving force of heterogeneous host-vector mixing in dengue transmission and the need to account for this phenomenon in individual based models[12].

What implications does this analysis of dengue transmission in 2 adjacent blocks have for dengue surveillance and control programs, which usually operate at the city or national level? One common detection-response action of dengue prevention programs is to apply vector control interventions (insecticides, larval habitat reduction) in the vicinity of dengue cases. We suggest that this is likely to be a fruitless activity during non-epidemic periods because clusters of cases are likely to be self-contained in pockets of susceptibility within areas where immunity pervades. Similarly, if city or nation-wide control programs use aggregate case numbers to evaluate early warning epidemic thresholds, this could be misleading if cases represent an intense, but geographically confined outbreak. Overall, our results dispute the existence of a direct causal relationship between the density of *Aedes aegypti* and dengue risk in endemic cities, but support the targeting of long-term vector control programs in economically marginal neighborhoods. These represent areas where high rates of water storage, rural-to-urban migration and high fertility rates are likely to coincide.

Our simulations demonstrate the need to reflect on the constituent processes involved in the *p*_*esv*_ parameter and the reasons why these might vary at the level of 2-block patches. External social vector exposure represents the intersection between human space-time allocation and exposure to infectious *A*. *aegypti* in each activity space[30], both of which are strongly linked to different aspects of urban social behavior. One behavior in particular, visiting the homes of social contacts, has been linked to dengue propagation between relatively isolated *A*. *aegypti* populations[22]. The probability of *A*. *aegypti* exposure in each visit will vary as a function of its duration[26] and as such, familiar clustering of dengue epidemics is commonly observed[43, 44], as we did in the PCR22 patch. Species with central-placed activity patterns demonstrate a strong reliance on social activity in a central area close to home[38], as evidenced in the space-time allocation patterns of residents of dengue endemic neighborhoods in Peru [36]. Stronger local social relationships can play an important role in dengue epidemics at the neighborhood level [23], suggesting that patches with high levels of *p*_*esv*_ are more likely to be embedded in neighborhoods with denser social networks among residents. Moreover, residents of lower income urban neighborhoods such as La Fachada are more likely to have stronger local social networks and greater preference for sharing local space [32, 45].

Accordingly, we suggest that viral introduction rates in the PCR22 patch are unlikely to have resembled the sporadic pattern observed in our simulations at *p*_*esv*_ = 0.001, especially given the consistently high levels of DF reporting in the La Fachada neighborhood in the 10 years prior to our surveys. Low residence times and a high proportion of the population below 5 suggest the possibility of high rates of recruitment of non-immune individuals. However, our simulations indicate that if viral introduction occurs too frequently, non-immune immigration and birth rates must be unrealistically high to support the susceptible densities required to generate PCR22 events in simulation years 11–20. Another explanation is that prior DEN2 introductions did not generate sufficient amplification in local mosquito populations due to the stochastic and heterogeneous host-vector mixing. However, our model showed that initial epidemics (years 1–10) occurred in virtually all conditions simulated, except at very lowest levels of *A*. *aegypti* production, an unlikely possibility given that the PCR22 patch had the highest density of *A*. *aegypti* positive containers. Given these results we suggest that cross-protection from dengue serotypes 1, 3 and 4 played an important role in maintaining the DEN2 susceptible densities in the PCR22 patch. There is growing evidence that dengue serotypes generate multi-year cross-immunity, shaping both larger scale dynamics and leading to sero-epidemiological “memory” and serotype-specific heterogeneity in transmission at smaller spatial scales [18, 19, 46, 47]. We suggest that cross immunity induced by prior transmission of dengue serotypes 1, 3 or 4 lingered in the PCR22 patch during the last DEN2 epidemics. This cross-protection is likely to have subsequently waned, leaving DEN2 susceptible densities high enough to allow for explosive transmission.

In conclusion, we demonstrate the potential for intense and focalized outbreaks of dengue in highly endemic areas. Although this observation occurred in a pair of blocks with entomological and demographic conditions conducive to susceptible recruitment and viral amplification, our simulation model indicated that social relationships of residents beyond the 2-block area necessarily played a critical role in maintaining DEN2 susceptible densities. We suggest that highly dense social networks at the neighborhood scale can constrain dengue transmission in certain blocks by generating too much local viral introduction and preventing the growth of susceptible clusters. We recommend that future field studies focus on space-time correlations between rural immigration, birth rates and social behavior. All three of these traits constitute fundamental mechanisms of human adaptation in heterogeneous environments and may change in predictable ways in response to the changing socio-ecological conditions of dengue endemic cities [48]. Future models should also consider the possibility for interactions between social networks and cross-protection between serotypes, whereby the latter may alleviates the impact of highly connected social networks on depleting susceptibility.

## Supporting Information

S1 Checklist STROBE Checklist. (DOCX)

S1 Code Agent-based model source code. (ZIP)

S1 File Supporting Information File. (DOCX)
