# Supplementary material for: Human Social Behavior and Demography Drive Patterns of Fine-Scale Dengue Transmission in Endemic Areas of Colombia
Source: PLoS One. 2015 Dec 14;10(12):e0144451. doi: 10.1371/journal.pone.0144451 (PMC4684369; doi:10.1371/journal.pone.0144451)
Supplement: S1 File — (DOCX) [file pone.0144451.s003.docx]

**Electronic supplementary material**

**Sensitivity and specificity of viral detection method**

RT-PCR based detection of dengue virus RNA in plasma or serum is a widely used diagnostic method that allows for early detection of infection and quantification of virus RNA concentrations well below the concentrations required for human to infect mosquitoes [[1-3](#_ENREF_1)]. Relatively high DEN2 concentrations during the first 2-3 days of illness have been associated with increased transmission to mosquitoes, with almost no transmission occurring after 5 days of illness [[2](#_ENREF_2)]. The commercially available kit that we used in this study could detect 100 viral copies/μL. In an independent validation it showed a sensitivity of 87% among patients within 3 days of onset of fever, that were later found positive for dengue IgM antibodies [[3](#_ENREF_3)]. Due to this short timescale of detectable viremia we cannot discard that high levels of infection had not been present in the nine surveys in which relatively low dengue prevalences were observed (Table 1). However, these short timescales also reinforces our conclusion, based on the PCR22 finding, that dengue transmission can be highly intense within a short space-time window.

In order to avert potential questions about specificity, all positive samples were sequenced. Cromatograms from the sequencing process showed single, well defined “peaks” for each nucleotide, discarding the presence of quasispecies, which might occur between viruses from the same individual. Nucleotide differences between samples were sufficient to discard cross contamination or unspecific amplification.

**Epidemiological context of PCR22 survey**

The survey in the PCR22 patch was carried out in the city of Armenia at a time with no reported DF cases in the neighborhood and approximately 6 months after the peak of the largest epidemic on record in the city (Fig S4). 2010 also saw the highest number of dengue cases reported in Colombia as a whole since record keeping began, with the vast majority of cases thought to be caused by DEN2, based on the isolates sequenced in the INS [[4](#_ENREF_4)]. DEN2 is also the most commonly identified dengue serotype in Colombia over the past 30 years [[4](#_ENREF_4)]. Moreover, the PCR22 event occurred in La Fachada, the neighborhood with the highest DF reporting (2001-2007) in Armenia, the city with the highest cumulative dengue incidence *and* total reported DF cases in Colombia during the same period (National Epidemiological Surveillance System of the INS).

**Simulation of daily household *A. aegypti* dynamics**

*A. aegypti* eggs, larvae and pupae develop in ephemeral household water-holding containers where human behaviors cause dramatic fluctuations in *A. aegypti* production at daily and weekly timescales, including both sudden population collapses and sudden bursts of pupation [[5-9](#_ENREF_5)]. Human behavior causes household or container level processes to heavily influence the dynamics of adult recruitment [[5-7](#_ENREF_5), [9-12](#_ENREF_9)]. *S*urveys commonly describe a highly aggregated distribution of pupae across houses, with certain houses with more persistent infestation; however, super-production is not spatially stable over time [[13](#_ENREF_13), [14](#_ENREF_14)]. In La Fachada we have carried out both a longitudinal study of 2-block patches[[15](#_ENREF_15)] and a study of the daily rate of production in infested vessels[[5](#_ENREF_5)]. These studies confirm that *A. aegypti* production concentrates in only a few houses at any given time[[15](#_ENREF_15)] and that bi-weekly processes play an important role in the dynamics of pupation [[5](#_ENREF_5)]. Armenia has little seasonal variation in rainfall and temperature and we found no clear seasonal patterns in the drivers of recruitment.

In the ABM newly-emerged host-seeking vectors randomly appear in a house based on its assigned pupal density, parameterized with data from 7 visits (2007-2009) to each house in each of 8 2-block urban patches (roughly 700 total households) in La Fachada. In each visit we inspected all water-holding containers, recorded whether or not they were infested with *A. aegypti* larvae or pupae, and counted all pupae. Every 15 days the model assigns each house a binary infestation status (absence or presence of an *A. aegypti* larval habitat) by sampling from one of the seven surveys carried out in each patch, giving 8 entomological scenarios. If a house of is assigned infestation, the number of pupae are randomly assigned from the counts across all containers in all 7 surveys of each patch (total of 720 infested containers found in La Fachada). Overall, the simulated production rates varied substantially across entomological scenarios (Fig 3), thereby providing a large variation in vector recruitment over which to analyze the model.

**Calculation of Levins niche-breadth**

Levins’ standardized niche-breadth [[16](#_ENREF_16)] of dengue in Armenia (B_t_) and was calculated for each 3-week interval in Armenia between 2001 and 2011, using the equation $B_{t}=(\sum_{i=1}^{R} p_{i,t}^{2})/R$, where R (resource) is the total number of DF-reporting neighborhoods in Armenia (we considered any neighborhood that reported at least 5 DF cases between 2001 and 2011 as potential resources for dengue) and *p_i,t_* is the proportion of total cases in 3-week interval *t* that resided in neighborhood *i.* *B_t_* measures the geographic spread of dengue in Armenia and increases in the from 0 to 1 as DF reporting becomes more evenly distributed across the city. The model was input with a 10-year time series of *B_t_*,  derived from the epidemiological surveillance data collected for each neighborhood in Armenia (2001-2011) (Fig S4). The time series of *B_t_* was repeated beginning in year 11 of the 20-year simulation.

**Classification and regression tree analysis**

We used classification and regression tree analysis (CART) to qualitatively describe variation in the drivers (model inputs) of simulated PCR22 events in years 0-5 as compared to years 11-20. CART analysis is a simple, graphical way to analyze complex datasets that may have multiple interactions and co-linearity [[17](#_ENREF_17)], and has been effectively used to study the dynamical behaviors of disease systems across a large parameter space [[18](#_ENREF_18)]. Trees (Fig S2) divide the data in a hierarchical manner using binary rules based upon single predictor variables. Threshold criteria chosen to partition the response variable into groups that are as homogeneous as possible. We used the Gini coefficient for binary response variables (occurrence of initial or endemic bursts) and least squares for continuous responses (correlation between outbreaks and persistence) (see [[17](#_ENREF_17)] for details on generation of splits). In order to avoid over-fitting, trees were pruned through a cross-validation method that minimized the amount of prediction error on testing data (not used in the construction of the tree) while also incorporating a penalty for increasing tree size [[17](#_ENREF_17)]. For example, between 0 and 5 years 100pupaefreq < 0.11 had 3797 model runs with a PCR22 event as compared to 14200 runs that did not, and this was the only split improved the predictive capacity over a CART model with no input variable included as covariates (Fig S2). For 11-20 years the number of PCR22 events was determined by a complex hierarchy of parameter inputs, with higher *100pupaefreq* influencing only within a subset of values for *mig* and *p_esv_.*

**Expanded sensitivity analysis of PCR22 events**

Overall, many parameters that might be expected to increase the force of dengue transmission had a more complex impact on PCR22 events in years 11-20, due to the negative feedback of infectious density on susceptible density. In general, PCR22 events were less sensitive to parameter variation at *p_esv_*=0.001, when susceptible density was not a limiting factor. Many parameters showed highest impacts on PCR22 events at *p_esv_*=0.01, notably vector production, survival, biting rate, dispersal and viral incubation period in mosquitoes. There was a clear interactive effect of net immigration of susceptible households and external social-vector exposure. At *p_esv_*=0.1 the system became highly sensitive to the rate of immigration of susceptible households, with a 50% rise over the baseline value (9 years^-1^) almost tripling the number of PCR22 events.

Beside immigration, surprisingly few of the human ecology parameters had clear effects on PCR22, with *p_esv_* increasing the level of variability in PCR22 events both across parameters and with 25% changes in each (FigS5a-c). Many of these parameters, including the rate of visiting local social contacts in adults and youth (*visit_a, visit_y*), the probability of having a social contact in any house (*link_p*) and the probability of social contact with close neighbors (*link_n*), controlled human-mediated viral propagation within the patch. Among the host-viral interaction parameters, increases in the human viremia period and the probabilities of human-to-mosquito and mosquito-to-human infection all increased PCR22 events at *p_esv_*=0.01. However at *p_esv_*=0.1 only mosquito-to human infection probability favored PCR22 events, and this effect occurred only at +50%. Extrinsic incubation period was the only parameter in the entire model that showed a strong monotonic relationship with PCR22 events at all *p_esv_* values.

Overall, variation in the vector ecology parameters had the largest impacts on PCR22 events, although these were far from monotonic or linear. Vector production had a strong positive effect on PCR22 events at *p_esv_* 0.001 and 0.01, but took on a humped shape at *p_esv_* 0.1. Vector survival showed a humped relationship with PCR22 events at all values of p_esv_ . When vector survival was too high, initial epidemics (years 1-10) likely became so explosive that susceptibility in the patch was decimated. Even at *p_esv_*=0.001, when susceptibility accumulated the fastest, increased daily survival probability from 0.9 to 0.96 prevented the occurrence of PCR22 events in years 11-20. Vector dispersal had an increasingly negative effect on PCR22 events as *p_esv_* increased and susceptible density became limiting.

**Correlation between PCR22 events and frequency distribution of outbreak size**

We determined the utility of our observation of 22 simultaneous dengue infections as a benchmark for evaluating the simulated dynamics. Overall there was a significant (p<0.01) positive correlation between the average number of simulated PCR22 events and the best-fitting power law exponent (*a*) for a given parameter combination. This correlation was weakest at *p_esv_*=0.001 (R^2^ = 0.23)*,* when the power law distribution did not fit the outbreak size distribution. However, PCR22 events explained 65% and 81 % of the variation in *a* when *p_esv_=0.1* and *p_esv_=0.01,* respectively (FigS6)*.* These results indicate assuming sufficient viral introduction, PCR22 events more readily occur in socio-ecological conditions that produce outbreak size distributions with a “fatter” tail.

**Supplementary Figures**

Figure A. Combined impacts of socio-ecological conditions on median daily fraction of humans susceptible to dengue infection 11-20 years post-invasion, stratified by external social-vector exposure, migration and vector production. Median is averaged across 1000 trials for each parameter combination. Legend in (a) applies to a-c. Other parameter values given in Table 2.


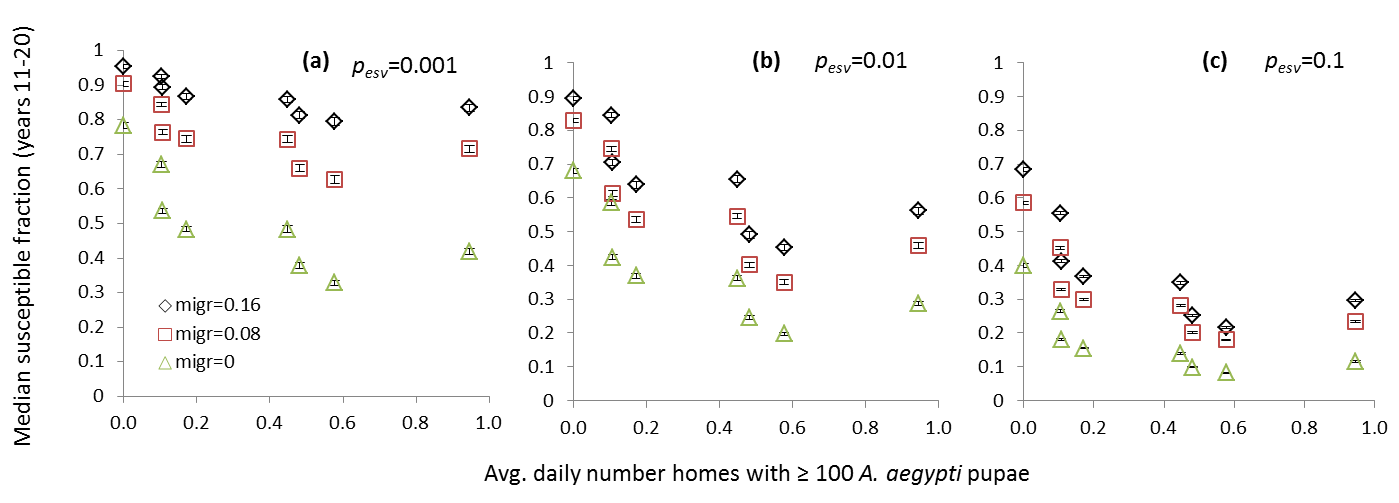


Figure B. Classification trees for the likelihood of a PCR22 event 0-5 years post invasion (a) and 10-20 years post invasion (b), determined over 1000 20-year simulation runs for each of 72 parameter combinations of *p_esv_*, *mig* and vector production (fertility varies randomly across runs and is classified into 4 quartiles). Each node of the tree is an inequality that splits the variable (model input) into two branches (in the left branch the inequality is true and in the right it is false). The split is produced at the midpoint of two discrete values of the variable input into the model. Below each node is the number of trials without/with the occurrence of a PCR22 event. The length of each branch is proportional to how much the node above improves the model. *100pupae freq* refers to the average daily number of houses that produce at least than 100 *Aedes aegypti* pupae. Models shown are the best-fitting trees pruned through a cross-validation procedure that stops the branching process when the addition of further splits no longer improved the model’s capacity to predict simulation outputs [[42](#_ENREF_17)].

(a)


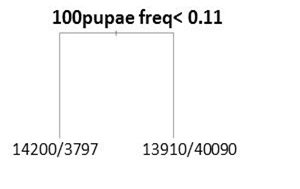


(b)


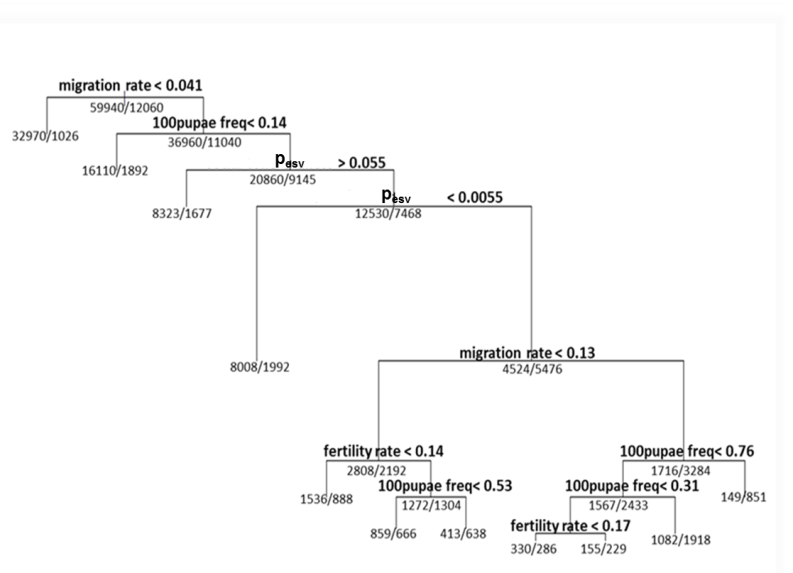


Figure C. Population age data collected in study patches located in Armenia, Bucaramanga (Bmanga) and Barranquilla (Bquilla); (no. PCR+ children/no. children tested)

Figure D. Levin’s Standardized Niche Breadth and triweekly reported DF cases (2001-2011) in Armenia, Colombia

R^2^ = 0.80

Figure E. Sensitivity of PCR22 events to model parameters, stratified by external social-vector exposure. Parameter descriptions and values given in Table 2.

**
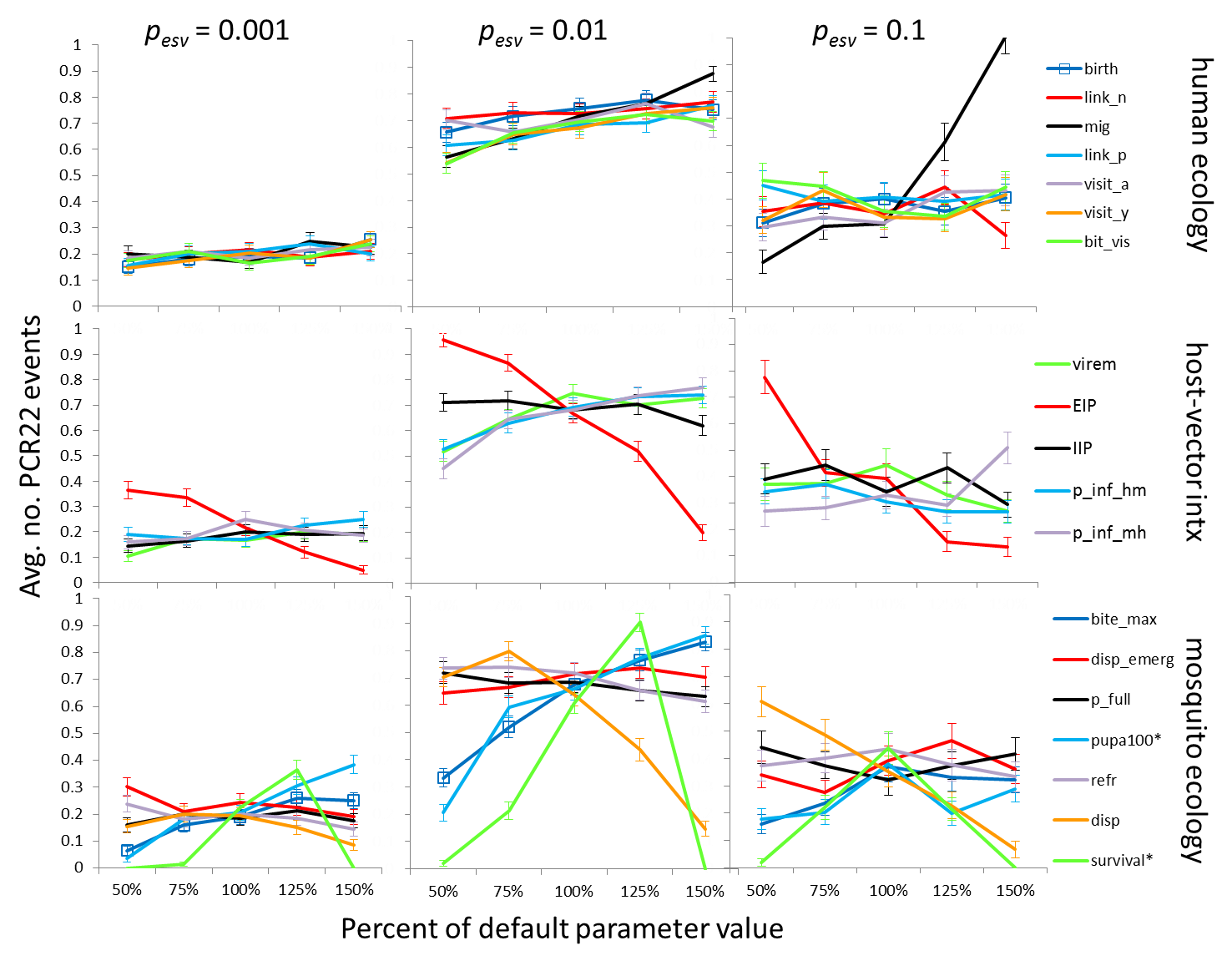
**

Figure F. Correlation between PCR22 occurrence and best-fitting power law exponent to simulated outbreak size distributions. Each data point represents 250 replicates of a unique combination of vector production (one of 8 study patches in La Fachada), migration and external social vector production (p_esv_). Parameter values given in methods section and Table 2.

Table A. Social and entomological features of study patches, listed in decending order of PCR prevalence (see Table 1)

| Patch ID | No. weekly visits received (residence if visitor) | | | No. A. aegypti pupae | Pupae per house | No. A. aegypti immature habitats | BI | Property size in neighborhood (m^2^) |
| --- | --- | --- | --- | --- | --- | --- | --- | --- |
|  | within neighborhood | within block | outside neighborhood |  |  |  |  |  |
| 1 | 2.1 | *1.3* | 1.1 | 20 | 0.34 | 11 | 9.8 | 53 |
| 2 | 2.1 | 2.0 | 4.3 | 30 | 0.61 | 6 | 1.4 | 95 |
| 3 | 1.6 | 1.1 | 2.7 | 201 | 2.48 | 16 | 5.9 | 38 |
| 4 | 1.5 | 2.1 | 2.7 | 0 | 0.00 | 1 | 0.4 | 95 |
| 5 | 2.4 | 1.0 | 1.9 | 77 | 1.57 | 11 | 5.9 | 92 |
| 6 | 0.9 | 1.1 | 1.5 | 20 | 0.42 | 3 | 1.9 | 72 |
| 7 | 2.8 | 2.5 | 1.7 | 17 | 0.33 | 5 | 2.9 | 92 |
| 8 | 0.5 | 0.5 | 1.1 | 17 | 0.39 | 5 | 4.4 | 95 |
| 9 | 3.3 | 2.5 | 1.2 | 0 | 0.00 | 2 | 1.7 | 92 |
| 10 | 3.5 | 3.8 | 3.0 | 4 | 0.04 | 6 | 2.0 | 95 |

1. Hue K.D.T., Tuan T.V., Thi H.T.N., Bich C.T.N., Anh H.H.L., Wills B.A., Simmons C.P. 2011 Validation of an internally controlled one-step real-time multiplex RT-PCR assay for the detection and quantitation of dengue virus RNA in plasma. *Journal of virological methods* **177**(2), 168-173. (doi:10.1016/j.jviromet.2011.08.002).

2. Nguyen N.M., Kien D.T.H., Tuan T.V., Quyen N.T.H., Tran C.N.B., Thi L.V., Thi D.L., Nguyen H.L., Farrar J.J., Holmes E.C., et al. 2013 Host and viral features of human dengue cases shape the population of infected and infectious Aedes aegypti mosquitoes. *Proc Natl Acad Sci U S A* **110**(22), 9072-9077. (doi:10.1073/pnas.1303395110).

3. De Paula S.O., de Melo Lima C., Torres M.P., Pereira M.R., Lopes da Fonseca B.A. 2004 One-Step RT-PCR protocols improve the rate of dengue diagnosis compared to Two-Step RT-PCR approaches. *Journal of clinical virology : the official publication of the Pan American Society for Clinical Virology* **30**(4), 297-301. (doi:10.1016/j.jcv.2003.11.004).

4. Mendez J.A., Usme-Ciro J.A., Domingo C., Rey G.J., Sanchez J.A., Tenorio A., Gallego-Gomez J.C. 2012 Phylogenetic reconstruction of dengue virus type 2 in Colombia. *Virology Journal* **9**. (doi:10.1186/1743-422x-9-64).

5. Padmanabha H., Soto E., Mosquera M., Lord C.C., Lounibos L.P. 2010 Ecological Links Between Water Storage Behaviors and Aedes aegypti Production: Implications for Dengue Vector Control in Variable Climates. *Ecohealth* **7**(1), 78-90. (doi:DOI 10.1007/s10393-010-0301-6).

6. Subra R. 1983 The regulation of preimaginal populations of Aedes aegypti L. (Diptera: Culicidae) on the Kenya coast. I. Preimaginal population dynamics and the role of human behaviour. *Ann Trop Med Parasitol* **77**(2), 195-201.

7. Subra R., Mouchet J. 1984 The Regulation of Preimaginal Populations of Aedes-Aegypti (L) (Diptera, Culicidae) on the Kenya Coast .2. Food as a Main Regulatory Factor. *Annals of Tropical Medicine and Parasitology* **78**(1), 63-70.

8. Gilpin M.E., Mcclelland G.A.H. 1979 Systems-Analysis of the Yellow-Fever Mosquito Aedes-Aegypti. *Fortschritte Der Zoologie* **25**(2-3), 355-388.

9. Jeffery J.A.L., Clements A.C.A., Nguyen Y.T., Nguyen L.H., Tran S.H., Le N.T., Vu N.S., Ryan P.A., Kay B.H. 2012 Water Level Flux in Household Containers in Vietnam - A Key Determinant of Aedes aegypti Population Dynamics. *Plos One* **7**(7), 6. (doi:10.1371/journal.pone.0039067).

10. Arunachalam N., Tana S., Espino F., Kittayapong P., Abeyewickreme W., Wai K.T., Tyagi B.K., Kroeger A., Sommerfeld J., Petzold M. Eco-bio-social determinants of dengue vector breeding: a multicountry study in urban and periurban Asia. *Bull World Health Organ* **88**(3), 173-184. (doi:10.2471/BLT.09.067892).

11. Getis A., Morrison A.C., Gray K., Scott T.W. 2003 Characteristics of the spatial pattern of the dengue vector, Aedes aegypti, in Iquitos, Peru. *The American journal of tropical medicine and hygiene* **69**(5), 494-505.

12. Hammond S.N., Gordon A.L., Lugo E.D.C., Moreno G., Kuan G.M., Lopez M.M., Lopez J.D., Delgado M.A., Valle S.I., Espinoza P.M., et al. 2007 Characterization of Aedes aegypti (Diptera : Culcidae) production sites in urban Nicaragua. *Journal of Medical Entomology* **44**(5), 851-860.

13. Morrison A.C., Gray K., Getis A., Astete H., Sihuincha M., Focks D., Watts D., Stancil J.D., Olson J.G., Blair P., et al. 2004 Temporal and geographic patterns of Aedes aegypti (Diptera : Culicidae) production in Iquitos, Peru. *Journal of Medical Entomology* **41**(6), 1123-1142.

14. TunLin W., Kay B.H., Barnes A. 1995 Understanding productivity, a key to Aedes aegypti surveillance. *American Journal of Tropical Medicine and Hygiene* **53**(6), 595-601.

15. Padmanabha H., Durham D., Correa F., Diuk-Wasser M., Galvani A. 2012 The Interactive Roles of Aedes aegypti Super-Production and Human Density in Dengue Transmission. *Plos Neglected Tropical Diseases* **6**(8). (doi:10.1371/journal.pntd.0001799).

16. Levins R. 1968 *Evotlution in Changing Environments.* Princeton, New Jersey, USA., Princeton University Press.

17. De'ath G., Fabricius K.E. 2000 Classification and regression trees: A powerful yet simple technique for ecological data analysis. *Ecology* **81**(11), 3178-3192. (doi:10.1890/0012-9658(2000)081[3178:cartap]2.0.co;2).

18. Cross P.C., Johnson P.L.F., Lloyd-Smith J.O., Getz W.M. 2007 Utility of R-0 as a predictor of disease invasion in structured populations. *Journal of the Royal Society Interface* **4**(13), 315-324. (doi:10.1098/rsif.2006.0185).

19. Domingo C P.G., Niedrig M, Cabrerizo M, Jabado O, Reyes N, Lipkin WI, Tenorio A. 2004 A New Tool for the Diagnosis and Molecular Surveillance of Dengue Infections in Clinical Samples. *Dengue Bulletin* **28**, 87-95.
